# p53 immunodetection of liquid-based processed urinary samples helps to identify bladder tumours with a higher risk of progression

**DOI:** 10.1038/sj.bjc.6602684

**Published:** 2005-07-05

**Authors:** E Piaton, J Faÿnel, A Ruffion, J G Lopez, P Perrin, M Devonec

**Affiliations:** 1 INSERM U.407, Université Claude Bernard Lyon I, Lyon, France; 2 Laboratoire d'Anatomie et Cytologie Pathologiques, Hôpital Edouard Herriot, Lyon, France; 3 Service d'Urologie, Centre Hospitalier Lyon Sud, Pierre Bénite, France

**Keywords:** p53, progression, urothelial carcinoma, liquid-based cytology

## Abstract

p53 could help identify bladder tumour cases with a risk of progression from superficial to invasive disease. Semiautomatic, liquid-based cytology (LBC) techniques offer an opportunity to standardise molecular techniques. The aim of our study was to investigate whether LBC could improve p53 immunolabelling, and to assess whether urinary p53 could have a prognostic value. Immunoreactivity for p53 was studied in 198 urine samples after treatment with the Cytyc Thinprep® processor. After antigen retrieval, cells were labelled with a monoclonal antibody that recognises both wild-type and mutant form of the p53 protein (Clone DO-7, Dako), 1/1000. Positivity for p53 was assessed in 17.2% of the cases. High-grade (G3) tumours were positive in 74.1% of the cases. Comparatively, low-grade (G1–2) urothelial carcinomas were positive in 23.5% of the cases. During a median follow-up period of 26 months, recurrence was observed in 52.9% of the cases with p53 overexpression, and in only 10.9% of negative cases (*P*<0.001). The progression rate was 35.3% of p53-positive cases *vs* 5.5% of p53-negative cases (*P*<0.001). Progression-free survival was significantly shorter in patients with p53 accumulation (*P*=0.007). In a multivariate analysis stratified on grade and stage, p53 was an independent predictor of overall survival (*P*=0.042). The results show that using Thinprep® LBC, p53 immunolabelling of voided urothelial cells allows most high-grade tumours to be detected and may help identify cases with a higher risk of recurrence and progression.

About 70% of superficial (TNM stage pTa–1) bladder urothelial carcinomas will recur in the 5 years following transurethral resection (TUR), and 10–20% will progress to muscle invasion (Saad *et al*, 2001). Patients treated for bladder cancer are therefore regularly followed up by cystoscopy and urinary cytology, but to date, no biological marker has gained wide acceptance in monitoring for tumour recurrence.

The p53 tumour suppressor gene is located on chromosome 17p. Mutations and loss of heterozygosity (LOH) commonly induce p53 inactivation, which correlates with protein accumulation in the nuclei of tumour cells. Numerous studies have shown a clear correlation between p53 gene mutation and the tumour stage and grade of bladder tumours (Brandau and Böhle, 2001). Deletions of 17p have been observed in more than 60% of patients with high-grade urothelial carcinomas, as well as in those who recur with muscle infiltration (Friedrich *et al*, 2001).

Since the initial data that reported a close correlation between p53 and prognosis, different and somewhat contradictory results have been published on tissue samples (Schmitz-Dräger *et al*, 2000).

In cytological samples, p53 overexpression has been shown to have only 23.5% sensitivity and 75.0% specificity in detecting bladder tumours (Righi *et al*, 1997), but in other series, sequence analysis of voided urine specimens compared with tissue samples showed 84.2% sensitivity and 96.8% specificity (Prescott *et al*, 2001). For some authors, p53 overexpression in the urine could aid in predicting prognosis and in identifying cases that are likely to progress from superficial to invasive disease (Wolf *et al*, 2001).

Liquid-based cytology (LBC) automatons using filtration and thin-layer deposition of cells have been developed to replace cytocentrifugation methods, owing to cell recovery capabilities and better cell morphology. In the urine, the use of the Thinprep® 2000 (Cytyc Corp., Boxborough, MA, USA) processor results in increased cellularity and marked reduction of debris, erythrocytes and crystals (Anagnostopoulou *et al*, 2000; Wright and Halford 2001; Piaton *et al*, 2002, 2004). Additionally, complementary techniques such as immunocytochemistry, DNA, RNA and protein molecular analysis can be successfully applied to Thinprep®-processed samples, thus allowing the impact of biomarkers in a variety of human malignancies to be studied (Tisserand *et al*, 2003).

Considering this background, the present study was undertaken to investigate (1) whether LBC could help study p53 overexpression in voided urothelial cells and (2) whether p53 overexpression in the urine could be linked to increased risk of recurrence and/or infiltration in bladder tumour cases.

## MATERIALS AND METHODS

The study group consisted of 198 urine samples collected from consecutive patients viewed at consultation in two departments of Urology in Lyon, France. The study was approved by the regional ethics committees. Samples were obtained from male and female patients at least 18 years old, either consulting for symptoms possibly indicating bladder cancer (mainly gross haematuria) or being followed up after complete TUR or bacillus Calmette–Guérin (BCG) immunotherapy for bladder urothelial carcinoma at least 1 month earlier.

The results of cystoscopy were recorded as positive (papillary growth or abnormalities in flat mucosa strongly suspicious for CIS), suspicious (irregular appearance in flat mucosa, not otherwise specified) or negative. A TUR was performed in every case of papillary bladder lesion, and mucosal abnormalities suspicious for CIS were evaluated by biopsies. Histopathological characterisation and grading used was that of the WHO/ISUP 1998 classification (Epstein *et al*, 1998) and the staging was performed according to the UICC TNM classification.

Whether on initial presentation or during the follow-up, voided or cystoscopically obtained urine specimens were fixed with an equal volume of Carbowax (20% polyethylene-glycol 1500 in 50% ethanol). The resulting samples were sent to the laboratory within 12 h, together with clinical and cystoscopy data.

### Preparation of LBC slides

After thorough homogenisation, the urine was processed according to the instructions for nonmucoid fluids provided by the manufacturer: samples were washed in phosphate-buffered saline and cells were resuspended in a Cytolyt® solution containing methanol, mucolytic and haemolytic agents. After 30 min at room temperature (RT), cells were centrifuged at 600 **g** for 10 min and resuspended in 45 ml PreservCyt® solution before being treated.

The Thinprep® 2000 automaton allows thin-layer cell preparations to be provided owing to a filtration process: after the TransCyt® filter has been plunged into the sample, it rotates at a high speed and facilitates cell and mucus dispersion. A vacuum is then applied to the filter, which collects cells on a 5 *μ*m porosity membrane. A software program allows a homogeneous deposition of cells until saturation. The TransCyt® filter is then inverted and a positive pressure allows cells to adhere to an electronegative slide. After insertion of another TransCyt® filter and of another slide, the whole procedure may be repeated until the entire sample has been treated.

In each case studied, the first slide obtained was stained with a hypochromic Papanicolaou procedure for cytopathologic evaluation. The second slide, when available, was used for p53 immunocytochemistry: cells were air-dried overnight, wrapped in metal foil and stored at −20°C.

### Urinary cytology and histopathology

Cytopathologic analysis was performed at low- and high-power fields. Normal, inflammatory, reactive and degenerative conditions of the urothelial component, as described by previous studies (Murphy, 1990; Bastacky *et al*, 1999) were considered as negative, as well as urothelial atypias of undetermined significance. Specimens in which neoplastic cells were recognised (grade 3, grade 2 clearly identified in the pathology report, strong suspicion for papillary low-grade tumours based on architectural and nuclear abnormalities) were considered as positive for urothelial carcinoma (Bastacky *et al*, 1999).

Histopathological results were separated into three groups: one positive for high-grade urothelial lesions (pTIS and G3 tumours, whatever their stage) according to the criteria of the WHO/ISUP 1998 classification (Epstein *et al*, 1998), one consistent with low-grade papillary tumours (G1–2, pTa–1 tumours) and one negative.

### Immunostaining methods

Before p53 immunolabelling, slides wrapped in metal foil were allowed to reach RT for 1 h. Cells were then fixed in 95% ethanol for 30 min. In cases where low cellularity did not allow slides to be prepared for p53 immunostaining, Papanicolaou slides were destained in 70% ethanol overnight (destained slides – see Table 3), rinsed in 70% ethanol and then fixed in 95% ethanol for 30 min.

Before labelling, slides were submitted to antigen retrieval for 15 min in 0.01 M citrate buffer, pH 6.0, at RT.

Cells were covered with a mouse monoclonal antibody to the p53 antigen (MoAb M 7001, clone DO-7, DAKO, Glostrup, Denmark) in a 1/1000 dilution for 1 h at RT in a dark wet chamber. This antibody recognises both wild-type and mutant forms of the p53 protein. Slides were treated in the Ventana GenII® processor (Ventana Medical Systems Inc., Tucson, AZ, USA) using a diaminobenzidine kit, and were slightly counterstained with Harris haematoxylin.

Negative controls were prepared by exclusion of the primary antibody. Known positive controls were included: slides were mixed with tissue sections of an archival bladder pT2 G3 urothelial carcinoma known for having 50–65% p53 nuclear reactivity. The tissue specimen also served as a negative control after exclusion of the primary antibody. The expression of p53 was studied in the three groups of cases. A urine sample was considered as positive for p53 when at least 10 nuclei were stained, provided at least 100 urothelial cells could be counted in the whole specimen.

For p53 immunohistochemical detection, tissue samples from the 73 formalin-fixed, paraffin-embedded TUR and biopsy specimens were stained as follows: representative 5 *μ*m sections were deparaffinised and rehydrated. A pretreatment microwave heating in 10 mM citrate buffer, pH 6.0, was performed before incubation with the primary antibody.

The sections were treated with the D0–7 antibody at 1/50 dilution for 45 min at RT. The reaction was revealed with the streptavidin–biotin complex method using an LSAB 2 Kit (DAKO S.A., Trappes Cedex, France). Tissue sections were considered as positive when at least 10% of nuclei were stained, whatever the intensity (*x*–*xxx*).

### Analysis of data

Time to recurrence was calculated from D0 (time of biopsies or TUR) to the date of the first documented recurrence or the last follow-up. Progression was defined as tumour recurrence at a higher stage (TNM stage pTa–1 with increased grade, or progression from pT1 to pT2 or more) and/or transition from G1–2 to G3, histologically documented metastases or death from bladder cancer.

Progression-free intervals were defined as the time between TUR and invasive tumour growth or G3 recurrence or end of the follow-up period. Fisher's exact test and *χ*^2^ test with Yates correction were used to measure the correlation between p53 accumulation and prognostic factors. Kaplan–Meier plots and the log-rank test were used to analyse the association of p53 with the time to recurrence and with progression. Two-sided *P*-values below 0.05 were considered to be statistically significant.

The Cox proportional hazards model was used for multivariate analysis of prognostic parameters (age, sex, grade, level of invasion and p53 status).

## RESULTS

The population studied was composed of 64 women and 134 men, 34–98 years old (mean age=67.3±11.7 years). There were 76 new patients consulting for symptoms possibly indicating bladder cancer and 122 patients followed up after TUR for bladder urothelial carcinoma.

There were 121 negative cystoscopy findings, 16 suspicious mucosal aspects leading to bladder biopsies and 61 typical tumour growths (Table 1). Overall, 61 bladder tumours were histologically diagnosed (27 G3 and 34 G1–2 urothelial carcinomas).

Urinary cytology was positive, high grade in 24 of 27 G3 urothelial carcinomas (sensitivity for high grade=88.9%) and showed tumour cells of various grades in 42 of 61 tumour cases (global sensitivity=68.9%), as shown in Table 2.

The mean volume of the urine samples obtained was 50.47±57.67 ml (range 30–200).

In the group demonstrating high-grade tumour cells in the urine, biopsies and TUR showed G3 urothelial carcinoma in 24 of the 32 cases with histopathological control (75.0%). High-grade tumour cells were evidenced in eight patients with low-grade urothelial tumours, probably revealing pTIS lesions not viewed at the cystoscopy level. These cases were also positive with p53 and four of eight cases (50.0%) recurred within a 24-month period.

### p53 immunoreactivity

LBC allowed a Thinprep® slide to be prepared for Papanicolaou staining in every case. However, after the first slide was obtained by the Thinprep® processor, the remaining material allowed a slide for p53 immunocytochemistry to be prepared in 158 out of 198 urine samples (79.8%).

The 40 remaining cases were treated as archival material: the Papanicolaou slides were destained and treated with the p53 MoAb as described in the Materials and Methods section. The expression of p53 was positive in two cases (5.0%), negative in 18 cases (45.0%) and unsatisfactory for evaluation in 20 cases (50.0%) as shown in Table 3.

Positivity for p53 was assessed in 34 of 198 urinary cytology cases (17.2%) in the whole series, including archival material in two cases. Positivity for p53 was assessed in 32 of 158 cases (20.3%) obtained after complete Thinprep® processing.

High-grade tumours were positive for p53 in urine in 20 of 27 cases (74.1%). All had positive, high-grade urinary cytology results.

**Figure 1:**
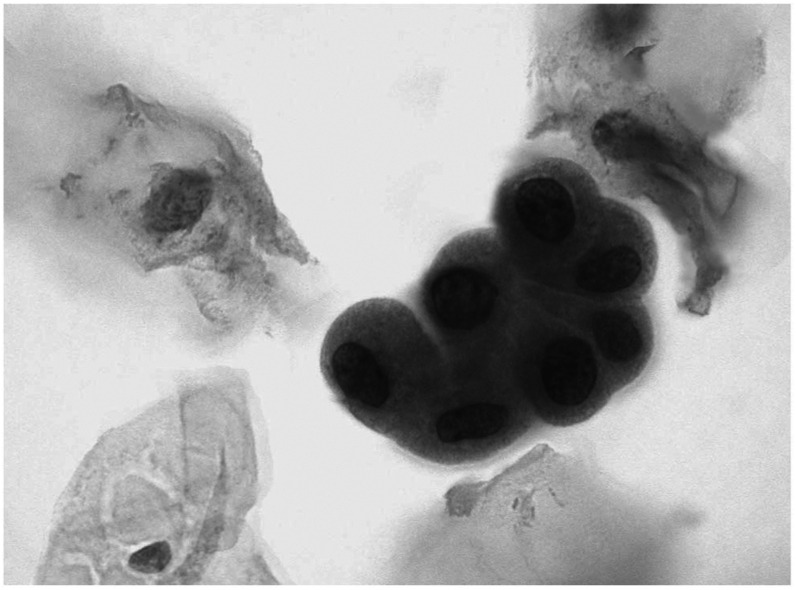
p53 immunostaining in a group of low-grade urothelial cells (× 400).

Comparatively, low-grade (G1–2) urothelial carcinomas were positive for p53 in urine in eight of 34 cases (23.5%), including six cases with urinary cytology findings suggesting low-grade proliferation (Figure 1).

The eight cases with low-grade urothelial tumours but positive, high-grade cytology findings showed positivity for p53 immunostaining.

Immunoreactivity for p53 was also tested on the tissue samples, in order to allow comparisons with other series: 22 of 27 high-grade cases (81.5%), and 16 of 34 G1–2 tumours (47.0%) showed positivity for p53. In comparison, the 12 negative tissue samples analysed were negative for p53 immunostaining.

### Prognostic relevance of p53 alterations in the urine

Most patients were evaluated at 12.0±4.2 and 24±5.6 months after D0 (time of initial evaluation). During a median follow-up period of 26 months (range 8–42 months), 36 of 198 patients (18.2%) had histologically proven bladder recurrence (median recurrence interval=13.7 months).

Recurrence was observed in 18 of 34 cases (52.9%) with p53 overexpression, and in only 12 of 112 negative cases (10.7%, *P*<0.001). Progression was noted in 18 of 146 valid cases (12.3%) with a median interval of 14.2 months. Of 18 (66.7%) progression cases, 12 were observed in patients with p53 positive immunolabelling.

Of 34 (35.3%) bladder tumours initially recorded as low grade (pTa–1 G1–2), 12 recurred within a 24-month period (Table 4). In the same period, progression was noted in six patients including four cases positive for p53: one pT1 G2 and one pT1 G3 bladder papillary tumours with CIS were evidenced at 12 and 18 months. Two pT1 G3 bladder tumours were diagnosed at 18 and 23 months. One case was a G3 upper urinary tract tumour diagnosed at 12 months in a patient with p53 unconclusive for evaluation at D0, and there was a pT2 G3 bladder tumour diagnosed at 20 months.

In patients with high-grade tumours at D0, recurrence at 24 months was assessed in 14 of 26 cases (53.8%) and progression was noted in 10 cases (38.5%).

**Figure 2:**
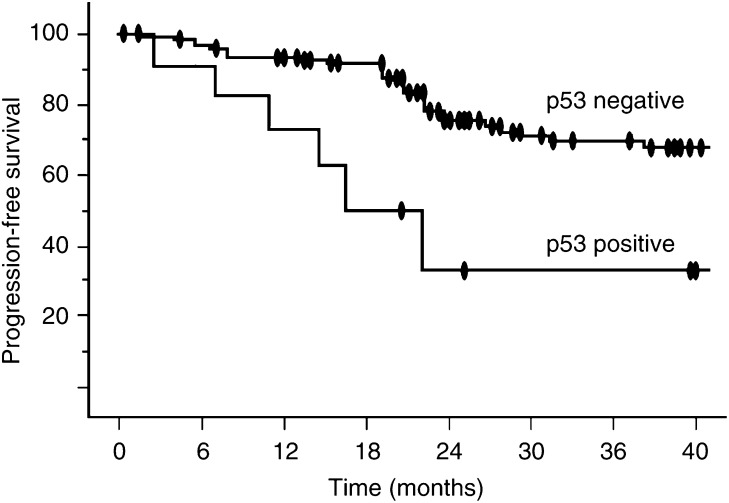
Kaplan–Meier progression-free survival curves in patients with p53-negative and p53-positive urine samples (*P*=0.007).

The progression rate was 12 of 34 (35.3%) p53-positive cases *vs* six of 112 (5.4%) p53-negative cases (*P*<0.001). Progression-free survival was significantly shorter in patients with p53 accumulation in voided urothelial cells (*P*=0.007, log-rank test, Figure 2).

In a multivariate analysis, p53 was an independent predictor of overall survival (*P*=0.042) when compared with age (*P*=0.898), sex (*P*=0.996) grade (*P*=0.097) and level of invasion (*P*=0.750).

## DISCUSSION

p53 is located on chromosome 17p13. The p53 gene is mutated in about 50% of human cancers, and nuclear accumulation of p53 protein demonstrated by immunohistochemistry is recognised as an independent prognostic factor for disease progression in most studies (Olumi, 2000). Most patients with allelic loss of 17p or p53 mutation have positive p53 immunoreactivity, as well as patients with LOH of the p16 locus located on 9p21 (Sourvinos *et al*, 2001). However, it has been shown that a fraction of patients with p53 overexpression has no genetic alterations, but has functional defects in the cell cycle regulation (Friedrich *et al*, 2001).

In bladder tumours, a number of studies have shown a positive correlation between p53 overexpression and mutation detection by DNA sequencing (Stadler *et al*, 2001; Hopman *et al*, 2002). However, p53 nuclear accumulation in the absence of gene mutation has also been noted.

The first studies of p53 alterations in the urine used a cloning approach followed by sequencing to confirm the presence of mutations (Sidransky *et al*, 1991). Thereafter, several reports have compared mutations in tissue samples with those found in the urine: p53 gene mutation in the urine has been shown to correlate with tumour recurrence or residual (Sachs *et al*, 2000). Voided urine specimens and bladder wash specimens have >90% accuracy in detecting p53 mutations compared with tumour tissue and show the same mutations after sequencing (Prescott *et al*, 2001).

Some authors have shown that a number of microsatellite alterations on p16, p53 and RB1 regions found in cytological urine specimens were not detectable in the corresponding tumour biopsies (Sourvinos *et al*, 2001). Such discrepancies suggest exfoliation of aggressive cell clones not sampled by biopsies, that is, originating from CIS areas better detected in cytological analysis.

However, only a few studies have been devoted to voided urine specimens, probably because attempts to use p53 as a diagnostic marker in urine have shown relatively low values (Righi *et al*, 1997). In the Righi series, the 23.5% sensitivity and 75% specificity reported render p53 less attractive than conventional urinary cytology for diagnosing bladder cancer.

Very few tumour markers (including Ki-67, uCyt+ and p53) have been recognised as having a prognostic impact in bladder urine specimens: a Ki-67 index over 20% may predict those pTa–1 G1–2 tumours that are likely to recur within 1 year of treatment (Gontero *et al*, 2000). Patients followed after TUR recur in 50% of cases when they have a positive uCyt+ assay despite negative cystoscopy in the year following urinary tests (Piaton *et al*, 2003).

In the literature, data on the prognostic value of p53 are controversial. The relevance of p53 in muscle-invasive, pT1 G3 bladder tumours is well documented, but the prognostic impact of p53 alterations in superficial, low-grade tumours remains uncertain (Friedrich *et al*, 2001; Wolf *et al*, 2001). Although some groups have shown that p53 is a significant predictor of bladder tumour progression (Schmitz-Dräger *et al*, 2000), others have concluded that it provides no prognostic information (Gontero *et al*, 2000; Friedrich *et al*, 2001).

There are a number of possible explanations for these contradictory results: first, some studies are based on small numbers of patients with various treatment modalities. Second, differences between laboratories may be related to the choice of p53 antibody, labelling protocol and scoring criteria. As stressed by Schmitz-Dräger *et al* (2000), standardisation of p53 immunolabelling is a matter of primary interest.

There is evidence that interactions between the antibody and the tissue might influence the outcome of the assay: p53 can be modified by phosphorylation (or acetylation/ubiquitination) at several sites, and the consequences of such phosphorylation for p53 function and epitope masking are still very poorly understood. DO-7, like DO-1, is sensitive to serine 20 phosphorylation, and this might underestimate the sensitivity of the assay (Dumaz *et al*, 2001). However, this aspect is never really discussed in review articles on p53 immunochemistry.

Although some studies have addressed the detection of mutated p53 in urine, none to our knowledge has studied the impact of LBC processing on p53 immunoreactivity. In the present series, LBC allowed a slide for p53 immunocytochemistry to be prepared in 79.8% of cases, and positivity for p53 was assessed in 32 of 34 cases (94.1%) after treatment by the Thinprep® processor.

In our opinion, the good results obtained are due to LBC technical improvements that allow pre-immunolabelling procedures to be standardised. In solid tumours such as in breast carcinomas, other authors have demonstrated that Thinprep®-processed samples allowed efficient DNA, RNA and protein recovery (Tisserand *et al*, 2003). In addition to improvements due to software-assisted filtration, the Thinprep® method allows unprocessed samples to be stored into the PreservCyt® solution, which maintains DNA, RNA and proteins suitable for molecular analyses even after several months of storage at 4°C or at −20°C (Tisserand *et al*, 2003).

In our series, p53 was positive in the urine in 46.7% of the tumour cases, and in 76.9% of the high-grade bladder tumours. Only 5.3% of cytologically negative cases were found p53 positive. Our results are comparable to those of Prescott *et al* (2001) in which 19 of 49 (38.8%) histologically confirmed tumours would have been detected by p53. However, no p53 mutation was detected in their negative cases.

More importantly, recurrence was observed in 52.9% of our cases with p53 overexpression, and progression was noted in 12.3% of the patients, 66.7% of progression cases being observed in patients with p53-positive immunolabelling. In the low-grade group, it is important to know whether p53 overexpression is linked to prognosis. From our results, in spite of the low number of patients, it is interesting to note that among eight cases with G1–2 bladder tumour and p53 positive in the urine, six (75.0%) have recurred and four (50.0%) have progressed within a 24-month period (Table 4).

Both data illustrate that p53 mutation detection has limited clinical utility for the detection of bladder tumours, but that voided urine specimens provide a good material for studying p53 in a prognostic attempt. Our results, combined with those of Tisserand *et al* (2003) obtained on cell lines and on breast cancer cells, show that Thinprep® LBC provides a very good material for molecular analysis, and that it could be considered as a technical standard for cytology-based molecular studies.

We conclude that owing to its negative impact on survival as demonstrated in this study, p53 in the urine might do more than play a simple role in identifying bladder tumour cases that may progress from superficial to invasive disease.

## Figures and Tables

**Table 1:** Cystoscopy and histopathology data in 198 patients

**Characteristic**	***n* (%)**
Negative cystoscopy (biopsy not performed)	121 (61.1)
*Suspicious aspects in flat mucosa*	16 (8.1)
Negative biopsy	10 (62.5)
Positive biopsy	6 (37.5)
pT2 G3	2
pTIS	4
	
*Positive cystoscopy (papillary lesions)*	61 (30.8)
Positive TUR	55 (90.2)
pTa G1–2	34 (61.8)
pTa G3	6 (10.9)
pT1 G3	9 (16.4)
⩾ pT2	4 (7.3)
pTa G2+pTIS	2 (3.6)
Negative TUR	2 (3.3)
TUR not performed	4 (6.6)
	
Total	198 (100.0)

**Table 2:** Correlation of urinary cytology (UC) with histopathology

	**Histopathology (%)**			
**UC results**	**G3**	**G1–2**	**Negative**	**Not performed**
Pos. high grade	24 (88.8)	8 (23.5)	—	2 (1.6)
Low grade	—	10 (29.4)	—	2 (1.6)
Negative	3 (11.1)	16 (47.1)	12 (100.0)	121 (96.8)
				
Total	27 (100.0)	34 (100.0)	12 (100.0)	125 (100.0)

**Table 3:** p53 immunostaining according to urinary cytology (UC) results

	**UC results (%)**			
**p53 status**	**High grade**	**Low grade**	**Negative**	**Total**
*Positive*				
Slides for p53	18 (52.9)	6^a^ (50.0)	8^b^ (5.3)	32 (16.1)
Destained slides	2 (5.9)	—	—	2 (1.0)
				
*Negative*				
Slides for p53	10 (29.4)	4 (33.4)	80 (52.6)	94 (47.5)
Destained slides	—	—	18 (11.8)	18 (9.1)
				
*Not conclusive*				
Slides for p53	4 (11.8)	2 (16.6)	26 (17.1)	32 (16.2)
Destained slides	—	—	20 (13.2)	20 (10.1)
				
Total	34 (100.0)	12 (100.0)	152 (100.0)	198 (100.0)

a Including two cases without histological confirmation.

b Including two false-negative UC cases with G3 bladder tumours.

**Table 4:** Progression according to initial histopathological data and p53 results in urine

			**Progression (%)**	
**Status at D0**	**No. (%)**	**Recurrence at 24 months**	**12 months**	**24 months**
*G1–2 bladder tumours*	34 (17.2)	12 (35.3)	—	—
p53 positive	8 (23.5)	6 (75.0)	0	4 (50.0)
p53 negative	8 (23.5)	2 (25.0)	0	0
p53 uc	18 (53.0)	4 (22.2)	2 (11.1)	0
				
*G3 bladder tumours*	27 (13.6)	14 (51.9)	—	—
p53 positive	20 (76.9)	10 (50.0)	4 (20.0)	4 (20.0)
p53 negative	6 (23.1)	4 (66.7)	0	2 (33.3)
p53 uc	0	—	—	—
				
*Negative histology*	12 (6.1)	0	—	—
p53 positive	0	0	—	—
p53 negative	12 (100.0)	0	0 (1 nr)	0 (1 nr)
p53 uc	0	0	—	—
				
*No histology*	125 (63.1)	10 (8.0)	—	—
p53 positive	6 (4.8)	2 (33.3)	0 (1 nr)	2 (33.3)
p53 negative	84 (66.7)	6 (7.1)	4 (17 nr)	0 (25 nr)
p53 uc	34 (26.9)	2 (5.9)	0	2 (5.8)
				
*Cumulated data*	198 (100.0)	36 (18.2)	—	—
p53 positive	34 (17.2)	18 (52.9)	—	12 (35.3)
p53 negative	112 (56.6)	12 (10.7)	—	6 (5.4)
p53 uc	52 (26.2)	6 (11.5)	—	6 (11.5)

uc=unconclusive for evaluation; nr=patient not reviewed.
